# A Qualitative Exploration of Trainee Nurse Practitioners' Experiences and Readiness to Provide Care for Borderline Personality Disorder in Primary Care

**DOI:** 10.1111/inm.70299

**Published:** 2026-07-06

**Authors:** Lisa Marie Riddell, Sophie Scott, Felicity Hasson

**Affiliations:** ^1^ Institute of Nursing and Health Research Ulster University Belfast UK; ^2^ Institute of Nursing and Health Research, School of Nursing & Paramedic Sciences Ulster University Derry/Londonderry UK; ^3^ Institute of Nursing and Health Research, School of Nursing and Paramedic Science Ulster University Belfast UK

**Keywords:** advanced nurse practitioner, mental health, personality disorder, qualitative research, trainee

## Abstract

To explore trainee Advanced Nurse Practitioners' clinical experience and professional preparedness in managing personality disorder presentations within primary care. A qualitative descriptive study using semi‐structured interviews and inductive thematic analysis. Between July and December 2024, 10 trainees enrolled at Ulster University completed online semi‐structured interviews. Data were analysed using Braun and Clarke's reflective thematic analysis framework, with rigour enhanced through triangulation. Four themes emerged: perceptions of patient attendance and engagement, workplace challenges, reflections on clinical experience and educational readiness. Participants described feeling underprepared for consultations, particularly around assessment, diagnosis and intervention. Limited training, time constraints, lack of clear pathways and systemic barriers were highlighted. Emotional strain and uncertainty often influenced clinical encounters. Participants emphasised the need for targeted continuing professional development, structured mentorship and crisis management training to build confidence and competence. Trainees face significant challenges in providing care for individuals with personality disorder in primary care. Missed opportunities in care were linked to limited training, systemic constraints and the emotional complexity of presentations. Strengthening psychiatric content in advanced practitioner curricula, alongside mentorship and organisational support, is essential for safe and effective practice. This study demonstrates the need for personality disorder‐specific education, access to interdisciplinary mentorship and policy‐level changes to support effective care in community settings. The study adhered to COREQ reporting guidelines. This study did not include patient or public involvement in its design, conduct or reporting.

## 1 Introduction

Globally, personality disorders are recognised as a significant mental health concern, affecting an estimated 6%–15% of the adult population (Volkert et al. 2018; Trull et al. 2019; Winsper et al. 2020). Personality disorders (PDs) are characterised by enduring patterns of inner experience and behaviour that deviate from cultural expectations and result in difficulties in emotional regulation, interpersonal relationships, self‐image, cognition and impulse control (American Psychiatric Association 1994, 2013; Herpertz et al. 2022). Historically, PDs have been classified into distinct diagnostic categories including borderline, antisocial, avoidant, narcissistic, obsessive‐compulsive, schizoid, schizotypal, paranoid, dependent and histrionic personality disorders (American Psychiatric Association 2013, 2022). More recently, classification systems have increasingly emphasised dimensional approaches to personality dysfunction and severity (Herpertz et al. 2022).

PDs are characterised by pervasive disturbances in cognition, affectivity, interpersonal functioning and impulse control, often leading to adverse outcomes such as mental and physical comorbidities, suicidality and premature mortality (Moran et al. 2016). Internationally and nationally, individuals diagnosed with PDs frequently seek initial assistance in emergency and primary care settings, making these departments critical points of contact for early intervention (Kent and Medway NHS and Social Care Partnership Trust 2017; National Institute for Clinical Excellence 2018; Vogel et al. 2019; Hain et al. 2025). These patients often present with crisis episodes, self‐harm and frequent emergency department visits, highlighting the need for effective management strategies (Bender et al. 2001; French et al. 2019).

Nurse practitioners are registered nurses with advanced training who can specialise in care for certain populations, such as psychiatric patients, and settings like primary care and emergency care (American Association of Nurse Practitioners 2022). However, research indicates that Advanced Nurse Practitioners (ANPs) and trainee ANPs working in these frontline clinical settings often lack the necessary training, confidence and clinical preparedness to effectively assess and manage PD presentations (Chadwick and Murphy 2019; Oliphant 2023). The limited integration of structured PD education within ANP and trainee ANP curricula exacerbates these challenges, contributing to increased reliance on specialist mental health services and variability in care delivery across settings (Morgan et al. 2022). Systemic barriers further hinder the effective management of PDs in frontline settings. These include limited consultation time, inconsistent access to mental health support and the absence of clear referral pathways, all of which contribute to fragmented care and poor outcomes (French et al. 2019).

While much of the existing literature has focused on the competencies and experiences of qualified professionals (Turi et al. 2023; Carotte et al. 2019), there remains a notable gap in understanding how well the future nursing workforce is being prepared to care for this complex patient group. Addressing this under‐researched area is critical to ensuring that trainee ANPs are equipped to deliver safe, effective and person‐centred care within primary care environments.

## 2 Background

Within both general practice and emergency care, ANPs play a crucial role in the holistic assessment, diagnosis and management of complex mental health presentations, including personality disorders (Mann et al. 2023; Ghatwal et al. 2021). However, despite the expansion of the ANP role, international literature highlights persistent inconsistencies in their training, scope of practice and clinical preparedness—particularly in the domain of mental health care (Wheeler et al. 2022).

Recognising the impact of PDs, international and national policies have been developed to improve mental health care provision. The Mental Health Declaration for Europe (World Health Organisation Europe 2005) emphasised the importance of public awareness, stigma reduction and improved access to mental health care. The Personality Disorder: No Longer a Diagnosis of Exclusion policy (National Institute of Mental Health England 2003) called for specialised PD services and enhanced training for healthcare providers. In Northern Ireland, the Personality Disorder Strategy: Diagnosis of Inclusion (Department of Health, Social Services and Public Safety 2010, Department of Health 2020, 2021) led to the establishment of the Regional Personality Disorder Service, providing a framework for improved care delivery (HSC Public Health Agency 2012, 2014). However, despite these initiatives, gaps remain in service provision, training and interdisciplinary collaboration (Dale et al. 2017).

While PDs have traditionally been managed by psychiatric services, there is increasing recognition of the role of primary care in their assessment and treatment (Morgan et al. 2022). ANPs, as key providers in primary and emergency care, are increasingly involved in the management of complex mental health presentations, including PDs (Mann et al. 2023). However, evidence suggests that many ANPs lack the necessary training, confidence and systemic support to manage these patients effectively (Oliphant 2023).

Diagnosing PDs in primary care is particularly challenging due to symptom overlap with other mental health disorders, the presence of comorbid conditions and the reliance on subjective clinical judgement (Herpertz et al. 2022). Patients often present with emotional dysregulation, impulsivity and recurrent crises, requiring skilled assessment and long‐term management strategies (Aslan et al. 2024). In emergency settings, time constraints and high patient turnover further complicate the accurate diagnosis and appropriate referral of PD patients (Shaikh et al. 2017).

Despite the increasing prevalence of PDs in primary care, research highlights significant gaps in ANP education and training. ANP curricula often lack structured content on PD assessment, diagnosis and management, leaving practitioners underprepared for complex psychiatric cases (Chadwick and Murphy 2019; Elvidge et al. 2024). Additionally, many ANPs report limited exposure to PD patients during clinical placements, leading to a lack of confidence in managing these cases independently (Kuczawski et al. 2024). The absence of interdisciplinary training further exacerbates these challenges, with limited opportunities for collaboration between primary care and mental health specialists (Fothergill et al. 2023).

Given increasing presentation rates in community settings—where up to one‐quarter of patients may meet the criteria for PD (Morgan et al. 2022)—there is a pressing need to ensure that the future nursing workforce is equipped with the necessary skills, confidence, and systemic support to manage these patients effectively.

## 3 The Study

### 3.1 Aim

The aim of this study was to explore trainee ANPs' clinical experience and professional preparedness in managing PD presentations within primary care settings.

## 4 Methods

### 4.1 Design

A qualitative descriptive design was employed to provide a comprehensive account of trainee ANPs' experiences in managing personality disorder (PD) presentations in primary care to capture participants' experiences with minimal interpretation. This study adopts an interpretivist paradigm to explore the subjective experiences and perceptions of trainee ANPs managing personality disorder in primary care. Interpretivism aligns with qualitative descriptive research, aiming to uncover nuanced human experiences without imposing predefined theories (Polit and Beck 2021).

This approach supports the collection of rich, practical insights relevant to curriculum and service development in primary and emergency care (Ayton et al. 2023). As noted by Holloway and Galvin (2016), it is well‐suited to healthcare research focused on real‐world application rather than theory building. By staying close to participants' own words, the design preserved authenticity while enabling systematic analysis through Braun and Clarke's (2022) thematic approach. This allowed inductive themes to emerge, reflecting real‐world practice, crucial in an under‐researched area where ANP perspectives remain limited (Herpertz et al. 2022; French et al. 2019).

This design aligns with the interpretivist paradigm, which focuses on understanding subjective human experiences (Holloway and Galvin 2016). It allowed the research to capture participants' narratives without imposing predetermined frameworks or seeking causal relationships (Ayton et al. 2023; Parahoo 2014). This approach was suitable given the limited existing literature in this specific area of advanced nursing practice. The study adhered to the COREQ checklist (Tong et al. 2007) to ensure transparency and rigour in design and reporting (see Appendix S1).

### 4.2 Study Setting and Sampling

The research was conducted within a single UK higher education institution. A non‐probability purposive sampling strategy was employed to recruit postgraduate nursing students enrolled in the ANP pathways for general practice and emergency care. Participants were enrolled on a UK Advanced Nurse Practitioner (ANP) master's programme and were undertaking advanced clinical practice training within General Practice or Emergency Care settings. In the United Kingdom, trainee ANPs are experienced registered nurses undertaking postgraduate education while developing advanced clinical competencies under appropriate clinical supervision. As part of their training, trainees manage patients presenting with a wide range of physical and mental health conditions, with levels of autonomy increasing throughout the programme. Eligibility was established through self‐declaration during recruitment. Participants were required to confirm that they had encountered and been involved in the assessment, management, referral or ongoing care of individuals presenting with personality disorder or suspected personality disorder within their clinical practice. Clinical encounters may have occurred under varying levels of supervision depending on stage of training and local service arrangements; however, all participants reported direct clinical exposure to this patient group.

This method was selected for its appropriateness in qualitative research where depth, rather than generalisability, is sought (Campbell et al. 2020). Participants had to have clinical experience in managing PD presentations and provide informed consent. Recruitment continued until data saturation was achieved, yielding a final sample of 10 participants with diverse clinical backgrounds. There were no participants who refused participation when approached.

### 4.3 Inclusion and Exclusion Criteria

In line with the purposive sampling approach, eligibility criteria were established. See Table 1 for an outline of the criteria applied in this study.

**TABLE 1:** Inclusion and exclusion criteria.

Inclusion criteria	Exclusion criteria
Enrolled in ANP general practice or emergency care pathway	Students without PD‐related clinical experience
Self‐reported direct clinical experience in the assessment, management, referral or care of individuals presenting with personality disorder or suspected personality disorder	Declined or unable to provide informed consent
Willing to participate in an interview	Participants unable to complete the interview process

### 4.4 Data Collection

Data were collected through semi‐structured online interviews using Microsoft Teams between July and December 2024 carried out by ANON alone. This method allowed for flexibility, participant comfort and accessibility (Sah et al. 2020). An interview guide, developed from a literature review and aligned with the study aim, contained 10 open‐ended questions (see Table 2). The guide was validated through expert review and a pilot interview. No repeat interviews were carried out.

**TABLE 2:** Questions from interview guide.

No	Question
1	How would you describe your understanding of personality disorders and their impact on patient presentation in an A&E OR GP setting?
2	What specific interventions or management strategies do you feel confident in implementing when caring for patients with personality disorder presentations in the A&E OR GP department?
3	Can you provide an example of a challenging interaction you've had with a patient who has a personality disorder? How did you approach the situation, and what did you learn from the experience?
4	In what ways do you believe the A&E OR GP department can better support patients with personality disorders, and how can nurse practitioners contribute to this support?
5	Describe a scenario in which you had to de‐escalate a situation involving a patient with a personality disorder. What steps did you take, and what was the outcome?
6	How do you ensure that you maintain empathy and non‐judgemental attitudes when working with patients who present with personality disorder symptoms in the A&E OR GP department?
7	What resources or professional development opportunities do you believe would enhance your ability to manage personality disorder presentations more effectively in the A&E OR GP setting?
8	Can you discuss a time when you collaborated with other healthcare professionals to provide comprehensive care for a patient with co‐occurring physical and mental health needs, including personality disorder symptoms?
9	How do you self‐assess your own emotional resilience and ability to cope with the challenging aspects of managing personality disorder presentations in the fast‐paced environment of the A&E OR GP department?
10	What strategies do you use to engage and involve patients with personality disorder presentations in their own care planning and decision‐making while in the A&E OR GP department?

All interviews were conducted by ANON, a female registered mental health MSc ANP trainee with 19 years' experience as a registered RMN working within community Adult mental health services. Verbal consent to record was reaffirmed prior to each session. Interviews were audio‐recorded, lasted 20–30 min and field notes were taken. A pilot interview was conducted following the same protocol as the main interviews to assess the clarity and flow of questions (Aziz and Khan 2020; Javadi and Zarea 2016). As no changes were required, and the data was relevant and rich, the pilot was included in the final analysis (Tate et al. 2023). Participants were assured confidentiality and instructed not to disclose identifiable patient information. Feedback on the findings has not been requested or provided to participants.

### 4.5 Data Analysis

Interview transcripts were produced via Teams' automated transcription, reviewed for accuracy by ANON, anonymised and assigned codes (see Table 3 for coding matrix). Data were analysed using Braun and Clarke's (2022) Reflexive Thematic Analysis framework, following their six iterative phases of familiarisation, coding, theme development, review, definition and reporting (Table 4), enabling inductive theme generation. This approach supported inductive, interpretative theme generation consistent with RTA's emphasis on researcher subjectivity as an analytic resource.

**TABLE 3:** Coding tree summary matrix.

Theme	Subtheme	Illustrative codes
Perceptions of patient attendance and engagement	Inconsistent presentation patterns	Patients attending in crisis; missed appointments
Perceptions of patient attendance and engagement	Stigma and misunderstanding	Fear of judgement; negative service experiences
Perceptions of patient attendance and engagement	Communication challenges	Mistrust; difficulty building rapport
Educational and professional readiness	Limited PD‐specific training	Lack of PD content in ANP curriculum
Educational and professional readiness	Inadequate clinical exposure	Few placements with PD patients
Educational and professional readiness	Confidence and competency gaps	Uncertainty in diagnosis and management
Support systems and professional networks	Need for mentorship and supervision	Lack of clinical supervision or mentors
Support systems and professional networks	Interdisciplinary collaboration	Limited interaction with specialist teams
Support systems and professional networks	Peer learning and informal support	Informal debriefing and peer guidance
Barriers to effective PD management	Systemic constraints	Short appointments; lack of continuity
Barriers to effective PD management	Organisational pressures	High workload; limited resources
Barriers to effective PD management	Policy and pathway gaps	Unclear referral procedures; regional variation
Emotional impact and professional vulnerability	Emotional labour of care	Feelings of burnout; working with trauma
Emotional impact and professional vulnerability	Fear of making mistakes	Risk anxiety; clinical pressure
Emotional impact and professional vulnerability	Coping strategies	Reflection; informal support seeking
Recommendations and future needs	Curriculum development	PD‐specific modules; communication training
Recommendations and future needs	Access to CPD and specialist input	Ongoing training; simulation learning
Recommendations and future needs	Structural reform	Trauma‐informed design; mental health integration

**TABLE 4:** Thematic analysis—six step approach.

Step	Process
1	Familiarisation with the data: reading and re‐reading transcripts to identify significant statements
2	Generating initial codes: highlighting meaningful excerpts and categorising them into preliminary codes
3	Searching for themes: grouping related codes into potential themes and subthemes
4	Reviewing themes: refining themes to ensure alignment with the data
5	Defining and naming themes: clearly identifying the essence of each theme and its relevance to the research aims
6	Producing the report

Coding and theme development were independently reviewed by two researchers (ANON and ANON), enhancing credibility through researcher triangulation and collaborative reflexive dialogue (Nowell et al. 2017). Supporting quotations illustrate each theme. Transcripts were not returned to participants for comment or correction due to resource constraints. Rigour was instead maintained through transparency in analytic decision‐making and sustained reflexive practice throughout the study (Moser and Korstjens 2018).

### 4.6 Ethical Considerations

Ethical approval was obtained from the School of Nursing and Paramedic Science Research Ethics Committee at the University. The study adhered to the Declaration of Helsinki (World Medical Association 2013) and GDPR guidelines. Participants received detailed study information, gave written informed consent and retained the right to withdraw. A distress protocol was followed, and participants were signposted to support services if needed. Risk disclosures were managed according to institutional safeguarding policies. Ethical Approval Number: FCNUR24‐035, Institution Name: Ulster University School of Nursing and Health Sciences, Ethics Filter Committee, Approved Date 8.05.2024.

### 4.7 Rigour and Reflexivity

The study maintained rigour through the trustworthiness criteria defined by Baillie (2015). Credibility was achieved using semi‐structured interviews and dual coding, which enhanced authenticity. Transferability was supported by providing rich contextual descriptions, enabling comparisons to similar settings. Dependability was demonstrated through the maintenance of an audit trail documenting research decisions and by checking data verbatim. Finally, confirmability was ensured by mitigating bias through anonymisation and researcher triangulation. The reflexive process supported neutrality, allowing themes to emerge from participant narratives rather than researcher assumptions.

## 5 Findings

### 5.1 Characteristics of Participants

The participant group consisted of 10 female trainee Advanced Nurse Practitioners (ANPs), all working in either General Practice (GP) or Accident & Emergency (A&E) settings (see Table 5). The majority (8 out of 10) were based in General Practice, while two participants worked in A&E. The length of time spent in their respective areas as trainee ANPs varied, ranging from 3 to 48 months, with an average duration of 12.9 months. The participants also had a broad range of professional experience, with years qualified as registered nurses ranging from 6 to 24 years, and an average of 14.9 years of experience. This diversity in clinical background, experience levels and training durations provides a valuable perspective on the challenges and learning needs associated with transitioning into the ANP role.

**TABLE 5:** Characteristics of participants.

Participant	Gender	Area	Time as trainee ANP	Years qualified
1	F	GP	48 months	24
2	F	GP	48 months	22
3	F	GP	12 months	13
4	F	GP	3 months	23
5	F	GP	3 months	8
6	F	A&E	3 months	17
7	F	GP	3 months	6
8	F	GP	3 months	12
9	F	A&E	3 months	9
10	F	GP	3 months	15

*Note:* Average time as trainee ANP = 12.9 months; average years qualified = 14.9 years.

Abbreviations: A&E, accident and emergency; GP, general practice.

In total, four themes were identified through the analysis of participant responses. These themes provide insight into the challenges, perceptions and preparedness of Advanced Nurse Practitioners (ANPs) in managing patients with personality disorder (PD). The themes include (1) perceptions of patient attendance and engagement; (2) challenges of the work environment; (3) reflections on clinical experience; and (4) educational and professional readiness. Each theme highlights key issues and includes direct participant quotes to illustrate the findings (see Table 3 for the coding matrix tree).

### 5.2 Perception of Patient Attendance and Engagement

Participants acknowledged a growing number of individuals presenting with mental health needs in primary care, often creating feelings of being overwhelmed and inadequately prepared. Many participants believed that individuals with personality disorder (PD) were not regular attenders in primary or emergency care settings, but this perception appeared to stem from limited awareness of the diverse and subtle presentations of PD. Instead, PD presentations were predominantly associated with acute crisis episodes—often when individuals had disengaged from specialist services or found them inaccessible. As one participant reflected. This highlighted a gap in recognition of chronic or milder manifestations of PD that might have allowed for earlier intervention.

We're seeing them when they're at breaking point—not before. (P8)

Patients with PD were often described as erratic and complex, complicating clinical engagement. Some participants admitted the impact of mental health stigma unconsciously influencing their attitudes toward these patients:

You try to be kind, but sometimes it's hard not to think they're just being difficult. (P5)

There was a strong desire to provide compassionate care, but this was often compounded by emotional strain and reduced empathy under pressure. Participants discussed experiencing an emotional burden when caring for individuals with PD. The fear of violence, stemming from perceptions of unpredictability in patient behaviour, heightened practitioners' sense of uncertainty. Some participants described feeling wary or anxious about managing risk:

It's emotionally draining when you feel like you're not making a difference. (P3)

You always wonder—is this going to escalate? (P6)

In response, some adopted more controlling or authoritative approaches during consultations to maintain safety and actively sought team input. Moreover, the invisible influence of stigma on clinical approaches was noted.

I'd rather call in a colleague early than deal with a situation alone. (P11)

Sometimes we assume they're being manipulative, when really they're just desperate. (P10)

Overall, participants painted a complex picture of caring for individuals with PD in primary care: marked by compassion, but challenged by systemic barriers, emotional labour and under‐preparation.

### 5.3 Challenges of the Work Environment

Participants described the emergency department (ED) and primary care environments as overwhelmingly negative spaces for patients with mental health presentations, including those with PD. The fast‐paced, high‐stimulus settings, combined with the lack of privacy, space, time and available mental health specialists, were consistently identified as major barriers to providing appropriate care. One participant captured the pace of work succinctly, whilst others noted the struggle in trying to provide compassionate care within restrictive time limits. As commented:

Appointments are very short, which can hinder providing holistic care. (P3)

It's hard to get them to focus on the main issue at the appointment, and I often go over the allocated time. (P8)

Many reflected that the clinical pressures often forced them into a task‐focused, rather than person‐focused, mode of care. In addition, the lack of quick access to specialist mental health staff was viewed as a critical weakness. Participants highlighted that the organisational drive for efficiency often overrode the quality of care. As reflected:

You want to listen, but you're watching the clock. (P4)

You can refer to mental health liaison, but they're overwhelmed too—it's not instant. (P9)

You're expected to manage everything quickly—mental health doesn't fit into a 10‐minute appointment. (P7)

This created internal conflict, with many practitioners feeling guilt and frustration about the sub‐optimal care they were able to offer. There was a strong call for system‐level reforms, including flexible scheduling, dedicated crisis management training and decision‐support tools to help manage complex PD presentations.

### 5.4 Reflections on Clinical Experience

Participants consistently reported a lack of confidence and uncertainty when communicating with and supporting individuals with PD. Many felt that their therapeutic communication skills were insufficient for dealing with this complex patient group. Several reflected that mental health issues often complicated the gathering of an accurate medical history. Whilst others described adapting their clinical approach, including slowing down physical examinations to help manage patient agitation. As stated:

It's hard to get a clear story—you end up guessing or ordering extra tests. (P2)

I tend to move slower, be more cautious—you don't want to escalate things by rushing. (P6)

A recurring theme was the lack of clear protocols for mental health presentations, leaving participants unsure about medication management and care planning. As a result, many ANPs reported a tendency to prioritise physical health concerns over mental health:

Once they're on multiple medications, I feel unsure about what to do next. (P9)

It's easier to deal with the chest infection than to start addressing their mental health. (P11)

Furthermore, fragmented care pathways and limited communication with mental health specialists contributed to a siloed approach to patient care:

We just add their name to the psych list and hope someone picks them up. (P8)

This left many feeling they were ‘patching over’ patient needs rather than addressing root causes. Although some participants had access to mental health practitioners within their practices, many commented on limited availability. However, the emotional toll of feeling helpless was highlighted. As commented:

We have a mental health practitioner, but they only work two days a week. (P6)

It feels scary that people need help, and I don't know how to give it. (P10)

Overall, there was strong recognition that improved training, resources and integration with mental health teams were urgently needed to support better clinical experiences and outcomes.

### 5.5 Educational and Professional Readiness

A predominant theme across participants was a significant gap in education and preparation regarding the management of personality disorder presentations. There was universal agreement that formal ANP training offered little to no structured content on PD or trauma‐informed care. Multiple participants expressed that they had no recollection of receiving any PD‐specific education resulting in some relied heavily on self‐directed learning to bridge these knowledge gaps:

No specific training that I can recall. I might have had a session but not sure. (P5)

I don't really have an understanding. I have mostly seen low mood but would like to know more. (P7)

I've mostly had to learn on my own, googling things or picking it up from colleagues. (P4)

There was a strong perception that the lack of formal mental health education undermined professional confidence:

I haven't been on any courses or training with regards to PD. I feel it's not an area I would be good at. (P11)

Participants expressed a particular need for more training in mental health assessment skills, risk management and building therapeutic relationships:

It would be really good to know how to undertake a thorough mental health assessment. (P12)

Placement experiences were described as a missed opportunity to build confidence, with limited mental health exposure during clinical training:

I've seen more colds and chest infections than anything mental health related. (P3)

Despite this, participants demonstrated high motivation for future professional development, suggesting integration of PD‐specific modules, mandatory de‐escalation training and resilience‐building programmes:

Short, informal awareness sessions or videos would be useful, especially for busy schedules. (P13)

Overall, the findings strongly highlighted that professional preparedness was significantly compromised by the lack of mental health‐focused education during ANP training. These left practitioners feeling ill‐equipped both academically and emotionally to manage the complexities of PD presentations.

## 6 Discussion

This study explored the clinical experience and professional preparedness of trainee Advanced Nurse Practitioners (ANPs) in managing personality disorder (PD) presentations within primary care settings. Thematic analysis revealed five consistent overarching themes: Perception of Patient Attendance and Engagement, Lack of Training and Educational Preparedness, Support Systems and Professional Networks, Barriers to Effective PD Management, and Personal and Professional Readiness to Manage PD. These themes reflect the complexity of managing PD in community settings and provide insight into how ANP education, support and systems influence clinical readiness. No minor themes or diverse cases were noted.

The findings should be interpreted within the UK healthcare context, specifically Northern Ireland, where personality disorder services are informed by Personality Disorder: No Longer a Diagnosis of Exclusion (National Institute of Mental Health England 2003), the Northern Ireland Diagnosis of Inclusion strategy (Department of Health, Social Services and Public Safety 2010) and National Institute for Clinical Excellence (2009) guidance. These policies promote community‐based, trauma‐informed and recovery‐oriented approaches to care. While service structures, referral pathways and advanced practice roles differ internationally, many of the challenges identified by participants including; limited training, uncertainty in managing personality disorder presentations, restricted access to specialist support and the emotional demands of care have also been reported internationally. Consequently, the findings may have relevance for nurse practitioner and advanced practice education beyond the UK setting.

### 6.1 Perception of Patient Attendance and Engagement

Participants reported that while mental health presentations were increasing, PD cases appeared under‐recognised or under‐reported in primary care settings. Several ANPs perceived that individuals with PD did not frequently attend services unless in crisis, often after disengaging from ongoing care. However, this may reflect a lack of awareness or confidence in identifying PD presentations rather than actual absence. Participants acknowledged that fragmented care pathways and time‐limited appointments often prevented thorough mental health assessments, resulting in a focus on immediate physical concerns.

This finding aligns with literature suggesting that individuals with PD often experience inconsistent engagement due to stigma, service fragmentation and symptom fluctuation (French et al. 2019; Morgan et al. 2022). The Royal College of Emergency Medicine (2021) notes that 45% of those who present with self‐harm in emergency settings have a PD diagnosis—highlighting missed opportunities for early engagement in primary care.

A proactive, trauma‐informed and relationship‐based approach may facilitate more consistent attendance and engagement. Training in motivational interviewing and personalised care planning could empower ANPs to develop therapeutic relationships with PD patients and contribute to continuity of care (National Institute for Health and Care Excellence 2018).

### 6.2 Lack of Training and Educational Preparedness

One of the most consistent findings was the reported lack of structured education on PD within the ANP curriculum. Participants expressed uncertainty regarding the assessment and management of PD, citing limited exposure and the need for more structured opportunities to build confidence.

This echoes previous research by Chadwick and Murphy (2019), who reported that ANPs often rely on informal learning and peer support when managing complex mental health presentations. The absence of formal training contributes to therapeutic pessimism and missed intervention opportunities (Bateman et al. 2007). Oliphant (2023) similarly highlighted that the generalist emphasis of many ANP programmes leaves clinicians ill‐equipped for managing psychiatric complexity in primary care.

To address this, PD‐specific modules, simulation‐based learning and supervised placements in mental health settings should be incorporated into ANP training. Kuczawski et al. (2024) and Rothwell et al. (2021) support the integration of interprofessional and experiential learning to build competence and reduce anxiety associated with PD care. Mentorship from mental health specialists was also identified by participants as a critical facilitator for skill development and emotional resilience.

### 6.3 Support Systems and Professional Networks

Participants described a strong reliance on specialist mental health colleagues, often due to gaps in their own training and the complexities associated with PD care. While collaboration was appreciated, it highlighted the need for greater confidence and autonomy among ANPs in managing these cases.

Literature supports the benefit of interdisciplinary networks, which can improve the coordination and quality of care (Tunks et al. 2023). However, overreliance on specialist services risks fragmenting care and reducing the scope of practice for ANPs. As noted by French et al. (2019), a more integrated model with shared learning and collaborative case reviews would enable ANPs to lead PD care confidently within primary care settings.

Participants in this study expressed a desire for regular peer supervision and reflective practice opportunities to process the emotional demands of PD work and prevent burnout. Embedding peer mentorship structures and reflective practice groups could enhance clinical decision‐making and emotional resilience among ANPs.

### 6.4 Strengths and Limitations of the Work

This study has some methodological limitations. First, the sample size was small and only representative of female perspectives, with participants recruited from a single educational site; therefore, the results may not be generalisable to other settings. Clinical experience of personality disorder presentations was also self‐reported and was not independently verified. Consequently, participants may have varied in the frequency, complexity, and level of autonomy associated with their encounters, reflecting differences in training stage and clinical placement experiences.

Future studies could benefit from a larger and more gender‐diverse sample to better understand how different practitioners experience and manage PD presentations. Second, as a qualitative study, the findings are inherently subjective and influenced by participants' personal experiences, biases and recall accuracy. The self‐reported nature of the data means that participants may have under‐reported or overemphasised certain challenges, potentially skewing the results. Additionally, interviewer bias could have influenced responses, particularly if participants sought to provide socially desirable answers rather than entirely candid reflections. There is a potential for bias as the lead interviewer was a trainee ANP; however, we sought to mitigate this by including an independent author's perspective in the analysis of the data.

Finally, the findings should also be interpreted within the context of the UK healthcare system and the Northern Ireland policy environment. Differences in healthcare structures, nurse practitioner regulation, mental health service provision and educational frameworks may influence the transferability of findings to other countries. However, many of the challenges described by participants, including limited preparation for mental health presentations, reliance on specialist services, and the need for enhanced supervision and mentorship, have been reported internationally. As such, the findings may offer valuable insights for nurse practitioner education and workforce development in comparable healthcare settings.

### 6.5 Recommendations for Future Research

This study underscores significant gaps in the preparation and professional support for Advanced Nurse Practitioners (ANPs) in managing personality disorder (PD) presentations, highlighting a critical area for improvement in nursing education and practice. The findings highlight the need for further research to promote rigorous scholarship and evidence‐based advancements in nursing science.

First further research is needed to evaluate the effectiveness of targeted educational interventions, including (1) continuing Professional Development (CPD) programmes tailored to PD management; (2) mentorship models that provide ongoing professional support; and (3) simulation‐based learning to improve ANPs' confidence and competence in managing complex cases. These studies may enhance ANPs' competence and preparedness in PD management, ensuring that evidence‐based educational strategies are implemented in clinical training.

Second, investigating the impact of interdisciplinary teams and collaborative learning environments will contribute to the development of sustainable models of care for PD management. Future research should focus on the effectiveness of joint training initiatives involving ANPs and mental health specialists and the role of multidisciplinary case reviews in improving decision‐making and care coordination. By examining these areas, research can inform best practices for fostering effective interprofessional collaboration in mental health management.

Finally, there is limited research on the long‐term impact of ANP training programmes on clinical preparedness and patient outcomes. Such research will help refine training curricula and professional development strategies to ensure lasting improvements in practice.

### 6.6 Implications for Policy and Practice

The study identified a marked absence of structured education in personality disorder (PD) management within ANP training pathways. Participants consistently reported uncertainty around assessment, risk formulation and appropriate interventions for PD presentations. This highlights the need to embed PD‐specific content into the ANP curriculum. Such content should not only cover diagnostic understanding but also crisis de‐escalation techniques and strategies for engaging individuals with fluctuating presentations. Structured education tailored to complex mental health needs will equip future ANPs with the knowledge and skills to manage these challenging cases more effectively and with greater confidence.

Many participants spoke of feeling emotionally overwhelmed when managing patients with PD, citing instances of uncertainty and frustration. These emotional responses reflect the psychologically demanding nature of working with this population and underscore the importance of fostering emotional resilience within ANP training. The inclusion of reflective practice sessions, supervised role‐play exercises and guided debriefing opportunities could enable ANPs to process challenging encounters, build coping strategies and develop empathy‐based communication. By preparing ANPs emotionally, in addition to clinically, programmes can enhance both practitioner well‐being and the therapeutic alliance with patients.

The findings also revealed that trainee ANPs frequently turned to specialist mental health colleagues for guidance and support, particularly when navigating unfamiliar or high‐risk scenarios. While collaboration is essential, this dependence also suggests a need to enhance generalist capacity. Promoting interprofessional learning opportunities such as joint supervision, shared case discussions and collaborative learning forums would support ANPs to develop their own expertise while benefiting from the knowledge of mental health specialists. These initiatives can bridge the confidence gap while fostering a team‐based approach to complex care.

Participants described the impact of systemic barriers, including unclear referral processes and the absence of formal support structures. Rather than recommending speculative solutions such as flexible scheduling, the study's findings support the introduction of clearly defined clinical pathways and accessible decision‐support tools for PD management. In addition, the development of localised protocols for risk assessment and escalation may help to reduce practitioner anxiety when managing patients in distress. Ensuring that ANPs have access to structured clinical guidance and regular supervision will promote safe and consistent care.

Finally, the study highlights the importance of mentorship and peer support as mechanisms for building clinical confidence and resilience. Participants valued support from experienced practitioners but lacked consistent access to such guidance. Establishing formal mentorship programmes within ANP education or workplace settings could ensure that trainees receive the supervision needed to develop confidence and maintain reflective practice. Peer support groups and facilitated case review meetings may also reduce feelings of professional isolation, providing a space for shared learning and mutual encouragement.

## 7 Conclusions

This study highlights critical gaps in the training and preparedness of ANPs to manage PD presentations in primary care settings. The themes identified—ranging from lack of training to systemic barriers—illustrate the need for targeted interventions to improve competence and confidence in PD care. By addressing these gaps through structured education, enhanced professional networks and systemic changes, ANPs can be better equipped to provide high‐quality, holistic care to individuals with PD. Moving forward, continued research and policy changes are needed to ensure that ANP training aligns with the realities of clinical practice, ultimately improving outcomes for both practitioners and patients.

### 7.1 Relevance to Clinical Practice

Personality disorder presentations present significant challenges and opportunities within primary care settings in Northern Ireland. Despite their high prevalence and impact on individuals' well‐being, personality disorders remain relatively overlooked and poorly understood within primary care. Addressing these challenges requires a concerted effort from healthcare organisations, policymakers and practitioners to enhance training, increase access to services and promote collaboration across disciplines. By improving recognition, assessment and management of personality disorders in primary care settings, we can better support individuals affected by these complex and enduring mental health conditions and improve their outcomes and quality of life. This study holds significant implications for healthcare policy, practice and education in Northern Ireland and beyond. By gaining a deeper understanding of the clinical experience and professional preparedness of trainee ANPs in managing personality disorder presentations within primary care settings, targeted interventions and training programmes can be developed to enhance their competence and improve the quality of care provided to individuals with personality disorders. Additionally, findings from this study may inform future curriculum development for ANP training programmes and contribute to the broader discourse on mental health service provision in primary care settings.

## Author Contributions

L.M.R. and S.S. made substantial contributions to the conception and design of the study and to the analysis and interpretation of data. L.M.R. led data collection. L.M.R. and S.S. drafted the manuscript and revised it critically for important intellectual content. All authors approved the final version to be published.

## Funding

The authors have nothing to report.

## Ethics Statement

Ethical approval was obtained from the School of Nursing and Paramedic Science Research Ethics Committee at Ulster University.

## Conflicts of Interest

The authors declare no conflicts of interest.

## Supporting information


**Appendix S1:** COREQ.

## Data Availability

The data that support the findings of this study are available from the corresponding author upon reasonable request.
